# The Preferable Binding Pose of Canonical Butyrylcholinesterase Substrates Is Unproductive for Echothiophate

**Published:** 2018

**Authors:** A. S. Zlobin, A. O. Zalevsky, Yu. A. Mokrushina, O. V. Kartseva, A. V. Golovin, I. V. Smirnov

**Affiliations:** Faculty of Bioengineering and Bioinformatics, Lomonosov Moscow State University, Leninskie gori, 1 , bldg. 73, Moscow, 119991, Russia; Shemyakin-Ovchinnikov Institute of Bioorganic Chemistry RAS, Miklukho-Maklaya Str., 16/10, Moscow, 117997, Russia; Institute of Molecular Medicine, I.M. Sechenov First Moscow State Medical University, Trubetskaya Str., 8, bldg. 2, Moscow, 119992, Russia; National Research University HSE, Myasnitskaya Str., 20, Moscow, 101000, Russia; Chemical Faculty of Lomonosov Moscow State University, Leninskie gori, 1, bldg. 3, Moscow, 119991 , Russia

**Keywords:** butyrylcholinesterase, echothiophate, organophosphates, QM/MM, metadynamics

## Abstract

In this paper, we, for the first time, describe the interaction between the butyrylcholinesterase enzyme and echothiophate, a popular model compound and an analogue of the chemical warfare agents VX and VR, at the atomistic level. Competition between the two echothiophate conformations in the active site was found using molecular modeling techniques. The first one is close to the mode of binding of the substrates of choline series (butyrylcholine and butyrylthiocholine) and is inhibitory, since it is unable to react with the enzyme. The second one is characterized by a significantly worse estimated binding affinity and is reactive. Thus, echothiophate combines the features of two types of inhibitors: competitive and suicidal. This observation will help clarify the kinetic reaction scheme in order to accurately assess the kinetic constants, which is especially important when designing new butyrylcholinesterase variants capable of full-cycle hydrolysis of organophosphorus compounds.

## INTRODUCTION

Butyrylcholinesterase (BChE) is an enzyme with broad substrate specificity: hence its significant importance as a basis for developing antidotes against organophosphorus poisons, such as the VX and VR nerve agents [1, 2]. Meanwhile, the kinetic scheme of the reaction catalyzed by cholinesterases is extremely complex, in particular, due to the presence of an additional peripheral anionic binding site (PAS). Close examination of the PAS for butyrylthiocholine, the characteristic BChE substrate, increases the total number of states of this enzyme in its kinetic scheme to eight [3]. If the substrate can irreversibly inactivate the enzyme due to the formation of a stable phosphorylated complex, the kinetic scheme can be even more complicated. Echothiophate is one of such substrates that both carry a choline moiety and have an inactivation potential. Echothiophate is a less toxic analogue of V-series chemical warfare agents and is used as a model organophosphorus compound to study the reactivity of butyrylcholinesterase and its inactivation-resistant modifications. In our study, the interaction between echothiophate and BChE was studied in order to evaluate whether the kinetic schemes earlier proposed for butyrylthiocholine can be used for it.

We decided to use molecular modeling methods, as they provide an atomistic insight into the ongoing events. Furthermore, they have previously proved effective in understanding the reaction mechanisms between BChE and some substrates [4] and even in rational modification of BChE and its transformation to the cocaine hydrolyzing enzyme [5].

## MATERIALS AND METHODS

Modeling of molecular docking was performed using the Autodock Vina software package [6]. The BChE structure with PDB ID 1XLW covalently conjugated to the product of phosphorylation by echothiophate, diethyl phosphate residue (DEP), was selected for docking analysis. DEP was removed, while the lacking residues V377-D378-D379-Q380 and C66 were recreated according to the structure of PDB ID 2XMD, since these structures are appreciably close to each other (the root mean square deviation (RMSD) calculated for all heavy atoms was 0.4 Å). The echothiophate structure was built using the Avogadro molecular editor [7]. The AutoDock Tools software was used to prepare the inbound files and process the results of docking [8]. The docking cell was centered so as to cover the entire binding pocket. All cell dimensions were 20 Å. For the sake of scanning efficiency, the exhaustiveness parameter was set to 64 and 20 independent replicas were performed. The enzyme remained rigid during docking, while the ligand had all degrees of freedom.

The starting configurations of BChE and the ligand were taken from the molecular docking procedure. Modeling of the metadynamics and data processing were carried out according to the procedure described in [9]. The Oγ(Ser198)-P(ECH) distance was used as a collective variable. The metadynamics potential, with a hill height of 2 kJ/mol and the adaptive width calculated using the diffusion criterion according to the previous 220 steps, was applied every 220 steps of molecular modeling. Three independent replicas were performed for each starting echothiophate binding pose.

## RESULTS AND DISCUSSION

**Fig. 1:**
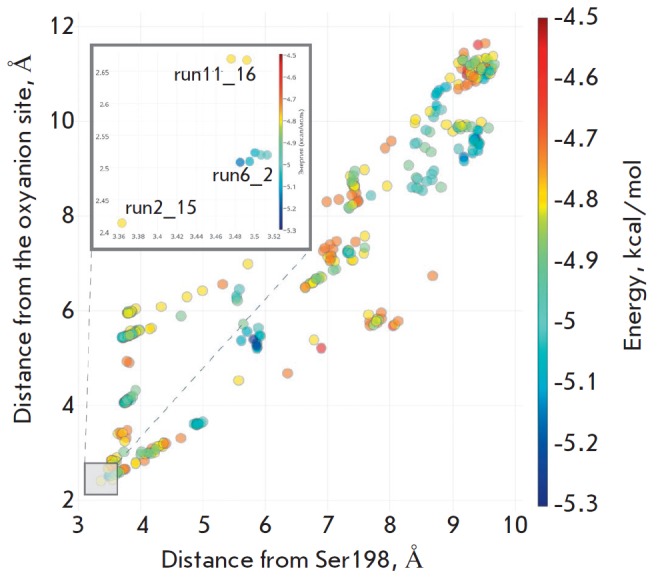
The results of docking of echothiophate into the binding pocket of BChE. The inset shows the best results from the lower left segment

The molecular docking studies were applied to search for the echothiophate binding position within the structure of human butyrylcholinesterase (PBD ID 1XLW). The echothiophate positions in the active site potentially capable of participating in the reaction (the ES state in the kinetic scheme [3]) were of specific interest to us. Therefore, we selected the main two metrics for the analysis of docking: (1) the distance between the oxygen atom of catalytic residue Ser198 and the phosphorus atom of echothiophate and (2) the distance between the center of mass of the oxyanion hole formed by backbone nitrogen atoms of the Gly116, Gly117, and Ala199 residues and the phosphoryl oxygen atom of echothiophate. The second metric was chosen because coordination of oxygen in the oxyanion hole is crucial for binding and positioning in the known mechanisms of the reaction [3]. Filtering by these criteria allowed us to single out the three best clusters of poses: run6_2, run2_15, and run11_16 (*Fig. 1*). According to the AutoDock Vina scoring function, the binding affinity of run6_2 pose is ~0.4 kcal/mol, better than that for the other two poses. Interestingly, the same arrangement of the choline moiety as in run6_2 is observed in the case of acetylthiocholine hydrolysis [4] and is presumably typical of ligands of a similar chemical nature. The interaction between the positive charge of the cationic choline group with the aromatic π system of Trp82 plays a key role in this case [10], while the Glu197 residue involved in catalysis has a smaller effect [10]. At the same time, this arrangement of the ligand leads to the leaving group - thiocholine - being located not on the line of nucleophilic attack.

**Fig. 2:**
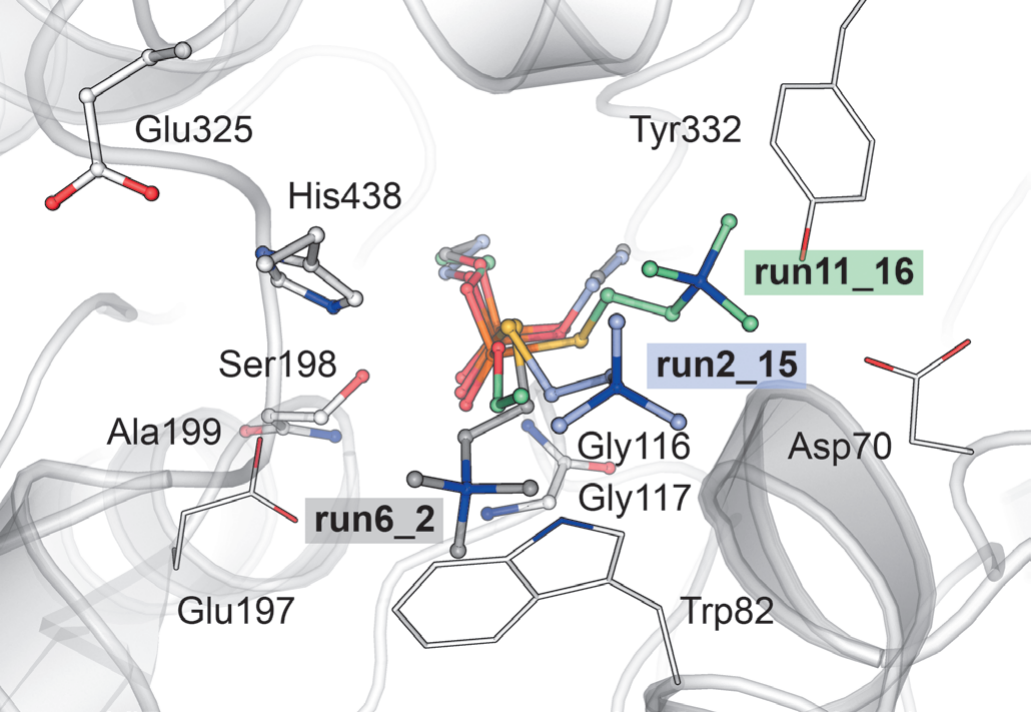
Three options for the starting position of the ligand. The residues included in the quantum system are indicated in the ball-and-stick model. Thin lines indicate the residues that bind the choline fragment. The carbon atoms of echothiophate in the binding variant run6_2 are shown in gray; run2_15, in blue; and run11_16, in green. The display of hydrogen atoms is omitted

In contrast, in the run11_16 position, the thiocholine leaving group is in line with the attacking Oγ Ser198 (*Fig. 2*) and the arrangement of ethyl groups is similar to that of the covalent conjugate in the 1XLW crystal structure [11]. The choline moiety, in turn, can electrostatically interact with the negatively charged Asp70 and the aromatic π system of Tyr332 within the peripheral anionic site (PAS) [10]. Previously, it was suggested that this position is the one most likely for echothiophate hydrolysis; the importance of the contact with the Asp70 residue was confirmed by a series of Asp70Gly and Asp70Lys mutants [12]. In this case, binding of the second substrate molecule in PAS is impossible. The run2_15 position is intermediate: the position of phosphate matches that in run6_2, while the choline tail occupies an intermediate position between run6_2 and run11_16 (*Fig. 2*).

We utilized hybrid quantum mechanics/molecular mechanics (QM/MM) modeling to estimate the reactivity of all three positions. In combination with metadynamics, the method designed to enhance sampling efficiency, this made it possible to estimate the energy barriers of the reactions [9].

**Fig. 3:**
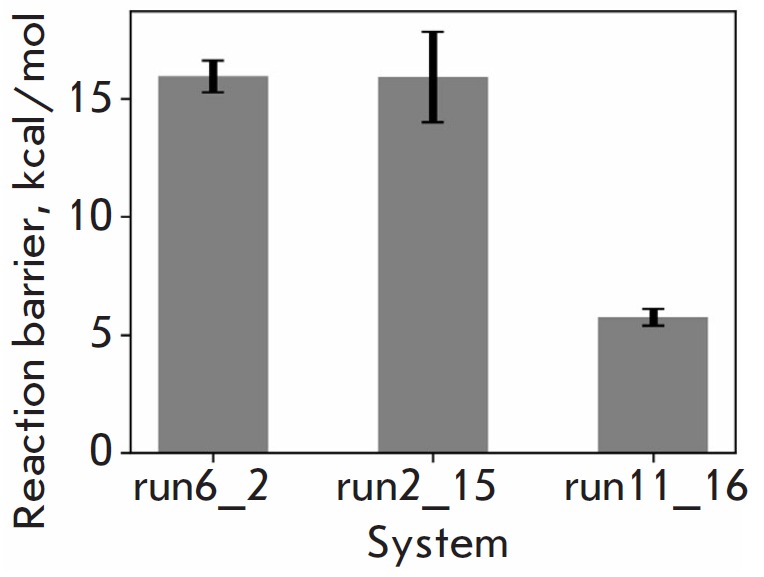
The estimated values of the reaction barriers for different starting positions. The average value and its error from three independent replicates are shown

The values obtained for run6_2, run2_15, and run11_16 are 15.9 ± 0.7, 15.9 ± 1.9, and 5.7 ± 0.4 kcal/mol, respectively (*Fig. 3*). These values are within the limits characteristic of enzymatic reactions in general, and are similar to those obtained in cases when the BChE reaction is studied with other substrates and using other computational methods [5].

However, the reaction barrier for the system run1_16 where the starting position of the ligand is such that the leaving group — thiocholine — is in line with the attacking oxygen Oγ Ser198 is noticeably lower, which makes the probability of a reaction from this position ~10^7^ times higher.

## CONCLUSIONS

We have used molecular modeling methods to reveal that there are two possible competing binding poses of echothiophate in the active site of butyrylcholinesterase. The first binding pose (the reactive one) had been predicted earlier. The second binding pose is inhibitory; it is close to choline substrates in terms of the binding mode and has a better binding affinity. Consideration of both of these binding poses will make it possible to refine the kinetic scheme of the reaction between echothiophate and butyrylcholinesterase, which is especially important in order to properly assess the kinetic constants when designing butyrylcholinesterase variants with phosphatase activity.

## Abbreviations

BChE: butyrylcholinesterase
ECH: echothiophate
RMSD: root-mean-square deviation
QM/MM: hybrid, quantum mechanics/molecular mechanics modeling
PAS: peripheral anion site

## References

[1] Ilyushin D.G., Smirnov I.V., Belogurov A.A. Jr., Dyachenko I.A., Zharmukhamedova T.I., Novozhilova T.I., Bychikhin E.A., Serebryakova M.V., Kharybin O.N., Murashev A.N. (2013). Proc. Natl. Acad. Sci. USA..

[2] Terekhov S.S., Smirnov I.V., Shamborant O.G., Bobik T.V., Ilyushin D.G., Murashev A.N., Dyachenko I.A., Palikov V.A., Knorre V.D., Belogurov A.A. (2015). Acta Naturae..

[3] Bevc S., Konc J., Stojan J., Hodošček M., Penca M., Praprotnik M., Janežič D. (2011). PLoS One..

[4] Chen X., Fang L., Liu J., Zhan C.G. (2012). Biochemistry..

[5] Zheng F., Xue L., Hou S., Liu J., Zhan M., Yang W., Zhan C.G. (2014). Nature Comm..

[6] Trott O., Olson A.J. (2010). J. Comp. Chem..

[7] Hanwell M.D., Curtis D.E., Lonie D.C., Vandermeersch T., Zurek E., Hutchison G.R. (2012). J. Cheminform..

[8] Morris G.M., Huey R., Lindstrom W., Sanner M.F., Belew R.K., Goodsell D.S., Olson A.J. (2009). J. Comp. Chem..

[9] Zlobin A., Mokrushina Y., Terekhov S., Zalevsky A., Bobik T., Stepanova A., Aliseychik M., Kartseva O., Panteleev S., Golovin A. (2018). Front. Pharmacol..

[10] Nachon F., Ehret-Sabatier L., Loew D., Colas C., van Dorsselaer A., Goeldner M. (1998). Biochemistry..

[11] Nachon F., Asojo O.A., Borgstahl G.E.O., Masson P., Lockridge O. (2005). Biochemistry..

[12] Masson P., Froment M.T., Bartels C.F., Lockridge O. (1997). Biochem. J..

